# Inter‐ and Intraspecific Competition in Invasive *Lactuca serriola* and Co‐Occurring Weedy Plant Species

**DOI:** 10.1002/ece3.70496

**Published:** 2024-10-29

**Authors:** Sohyun Woo, Tae‐Min Kim, Yousuk Kim, Seorin Jeong, Eunsuk Kim

**Affiliations:** ^1^ School of Earth Sciences and Environmental Engineering Gwangju Institute of Science and Technology Gwangju Korea

**Keywords:** competitive intensity, interspecific competition, intraspecific competition, invasive species, native weedy plants, nutrient

## Abstract

While invasive success of alien plant species is often attributed to their superior competitive abilities, it is also suggested that competitive ability depends on the target species of competition and resource availability. In addition, it remains unclear whether invaders and co‐occurring plants in the introduced area exhibit distinctive inter‐ and intraspecific competitive intensities. This study aimed to evaluate the competitive ability of a successful invader, *Lactuca serriola*, through a combination of field surveys and a growth chamber experiment. First, we assessed biodiversity and the biomass of co‐occurring plants in both *L. serriola*‐invaded and uninvaded plots across nine sites in South Korea. Subsequently, a pairwise competition experiment was conducted between *L. serriola* and three weedy plant species commonly found in the invaded plots, *Chenopodium album*, *Erigeron canadensis*, and *Oenothera biennis,* under differential nutrient levels. Diversity indices of plant communities and the biomass of most co‐occurring plants showed no significant difference between invaded and uninvaded plots. *L. serriola* and testing weedy plants exhibited mutually negative effects on biomass when grown together in the same pot, with the intensity of interspecific competition being comparable across nutrient treatments. Notably, intraspecific competition of *L. serriola* was weaker than testing weedy plants, particularly manifest in the high‐nutrient treatment. The results of both field and growth‐chamber studies demonstrated that *L. serriola* was not a particularly strong competitor compared to its neighboring weedy plants. Its successful invasion can be partially attributed to its weak intraspecific competition intensity, which potentially facilitate successful establishment with high density.

## Graphical abstract

Interspecific competition between the invasive *L. serriola* and co‐occurring weedy plants was similar, but *L. serriola* exhibited weaker intraspecific competition, especially in high‐nutrient conditions. This weaker intraspecific competition may help *L. serriola* establish high‐density populations, contributing to its successful invasion despite not being a strong competitor against other weedy species.

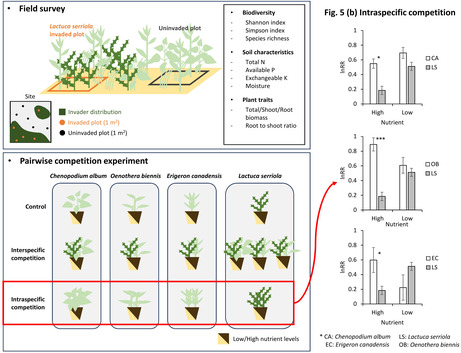

## 1 Introduction

Biological invasions are a growing concern amid ongoing global changes (Pyšek et al. 2020), prompting significant attention to uncover the determinants of invasion success (van Kleunen, Bossdorf, and Dawson 2018). The success of invasive alien plants is often attributed to their superior competitive abilities over plants inhabiting the introduced areas (Gaertner et al. 2009; Sakai et al. 2001; Vilà and Weiner 2004). As a consequence of their high competitive ability, invasive alien plants are predicted to detrimentally impact invaded ecosystems through competitive exclusion or replacement, possibly leading to a decrease in species diversity (Baker 1965; Pyšek et al. 2012; Roy 1990; Vilà et al. 2011).

While the hypothesis of superior competitive ability has been proposed, empirical studies have shown that some invasive alien plants exhibit inferior competitive abilities (Corbin and D'Antonio 2004; McGlone et al. 2012; Tesfay, Blaschke, and Kreyling 2023). For instance, several studies have emphasized that outcomes of competitive interactions likely depend on the plant species being tested, suggesting that the competitive ability of invaders may be higher than that of rare plants but similar to weedy or dominant plants (Dawson, Fischer, and van Kleunen 2012; Vilà and Weiner 2004; Zhang and van Kleunen 2019). Notably, invasive alien plants often possess traits characteristic of weedy species and successfully invade habitats in the introduced area where weedy plants dominate. Comparing the competitive abilities of invasive alien plants to those of dominant weedy species in the introduced areas can provide insights into the role of competitive ability in the invasive success of alien plants.

When evaluating the competitive ability of invasive plants, most studies focus on interspecific competition between invasive alien plants and plants in the introduced area (Gioria and Osborne 2014; Vilà and Weiner 2004). However, it should be noted that the outcome of competitive interactions depends on both interspecific and intraspecific competition (Hart, Freckleton, and Levine 2018). Plant species with weak interspecific competitive ability can coexist with stronger competitors if they cluster with conspecific individuals, benefiting from reduced intraspecific competition (Wassmuth et al. 2009). Considering the contribution of intraspecific competition to the outcome of competitive interaction, both intraspecific and interspecific competition should be taken into account when evaluating the competitive ability of invasive species (Zhang and van Kleunen 2019).

The availability of resources has been recognized as a key factor influencing the outcomes of interspecific competition between invaders and co‐occurring species. In highly fertile environments, rapid resource uptake and high resource use efficiency is considered as a primary driver of successful invasions (Goldstein and Suding 2014; Leffler, Monaco, and James 2011; Liu, Yang, and Zhu 2018; Schoenfelder et al. 2010). Conversely, natives often outperform invaders under low‐nutrient conditions (Daehler 2003), probably due to higher tolerance to low resource conditions (Catford et al. 2019; Funk 2013; Seabloom et al. 2003). Given the influence of nutrients on competition outcomes, it is necessary to examine the intensity of both interspecific and intraspecific competition in the context of nutrient availability.

*Lactuca serriola* (prickly lettuce) is a winter or summer annual herbaceous weed that originated in Mediterranean (Lebeda et al. 2004) and has invaded many regions worldwide, including northern Europe, Australia, and North America (Chadha and Florentine 2021; Hooftman, Oostermeijer, and Den Nijs 2006). *L. serriola* was first reported as an introduced alien summer annual in South Korea in 1978 (Kim et al. 2013). Since then, it has rapidly expanded its range and currently occurs throughout the country. It can grow up to 1.5 m high and occurs in open habitats like roadsides and abandoned agricultural fields (Weaver and Downs 2003). In introduced areas, *L. serriola* is known to inhabit plant communities composed of annual or biennial weedy species, such as *Erigeron canadensis*, *Capsella bursa‐pastoris*, and *Bromus tectorum* (Amor 1986; Hooftman, Oostermeijer, and Den Nijs 2006). Remarkable resistance to environmental stresses like drought is suggested as an attribute contributing to its invasive success (Chadha and Florentine 2021; Jeong et al. 2021; Werk and Ehleringer 1986), but the competitive ability of *L. serriola* and its role in the invasion remain unknown.

Here, we assessed the competitive ability of *L. serriola* through both field observation and a manipulative experiment. To evaluate its competitive effect, we compared biodiversity and biomass of co‐occurring plant species between invaded and uninvaded field sites. Additionally, we conducted a pairwise competition experiment in a growth chamber environment to evaluate interspecific and intraspecific competition of *L. serriola* and co‐occurring plant species. Nutrient treatments were applied to plants to examine competitive abilities under varying resource conditions. We measured two functional traits, the root‐to‐shoot ratio (RS ratio) and specific leaf area (SLA), to assess their contribution to the competition ability.

Specifically, we addressed the following questions: (1) Do invasive *L. serriola* negatively affect recipient plant communities and the growth of co‐occurring plant species? (2) Do *L. serriola* and co‐occurring plant species exhibit different intensities of inter‐ and intraspecific competition? (3) Do the outcomes of competition depend on nutrient availability?

## 2 Materials and Methods

### 2.1 Study Sites, Field Survey, and Sample Collection

**FIGURE 1:**
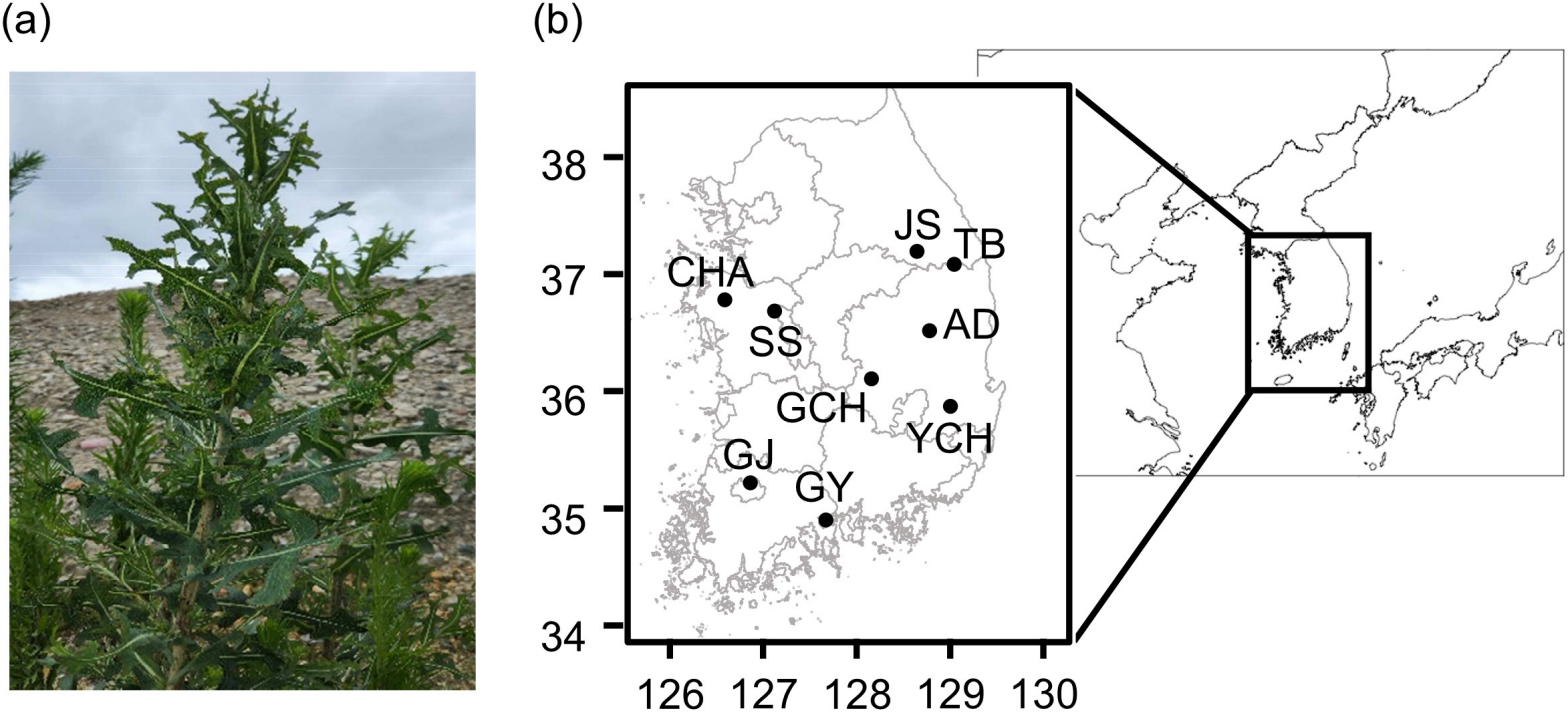
A photograph of *Lactuca serriola* (a) and the study sites where vegetation surveys were conducted (b). Latitude and longitude of each site are given in Table S1. AD, Andong, Gyeongsangbuk‐do; CHA, Cheonan, Chungcheongnam‐do; GCH, Gimcheon, Gyeongsangbuk‐do; GJ, Gwangju; GY, Gwangyang, Jeollanam‐do; JS, Jeongseon, Gangwon‐do; SS, Seosan, Chungcheongnam‐do; TB, Taebaek, Gangwon‐do; YCH, Yeongcheon, Gyeongsangbuk‐do.

Based on distribution information of *L. serriola* provided by the National Institute of Ecology, we randomly selected nine sites (> 200 m^2^) in South Korea (Figure 1, Table S1). In all sites, *L. serriola* showed a cover‐abundance scale of 7 or higher (Westhoff and Van Der Maarel 1978). These sites comprised fallow fields, vacant lots, or roadsides. As *L. serriola* is categorized as an ecosystem‐disturbing wildlife species under the Biodiversity Conservation and Utilization Act in South Korea, we obtained appropriate study permits from local authorities for the transportation, storage, and cultivation of plant materials.

Vegetation surveys were conducted from June to early August 2022. At each site, we randomly established five 1 × 1 m^2^ plots as invaded plots, with each plot containing 3–48 *L. serriola* individuals. Additionally, five 1 × 1 m^2^ uninvaded plots containing no *L. serriola* individuals were established nearby. All plants within the plots were identified following Lee (2003), and the individual counts of each species were recorded. Species richness, Shannon index, and Simpson index were calculated for each plot using *vegan* package in R software 4.0.3 (R Foundation for Statistical Computing, Austria). The volumetric water content of soil in each plot was measured using a Hydrosense II (Campbell Scientific, Utah, USA) in the field. Topsoil samples were taken near each plot to avoid disturbance within the plots, resulting in a total of 10 soil samples collected at each site. Soil characteristics were analyzed by CheilLab Inc. (Seoul, Korea), including total nitrogen using the Kjeldahl method (Kjeldahl 1883), available phosphorus using the Lancaster method (Cox 2001), and exchangeable potassium using the ammonium acetate method (Gavlak et al. 2003).

Nine plant species were found in both *L. serriola*‐invaded and uninvaded plots across the testing sites (Table S2). At each site, three dominant plant species that appeared in both invaded and uninvaded plots were selected based on the number of plant individuals present in the plots (Table S3). In late August and September 2022, we revisited study sites and collected up to three individuals of each selected species from each plot, resulting in a total of 14 to 30 plant individuals per species at each site. The number of individuals collected for each species is given in Table S3. Collected samples were brought to the laboratory, carefully washed with water, and then oven‐dried at 65°C for 72 h to measure their dry weight. While *Bromus japonicus* and *Vicia villosa* were dominant species at the Jeongseon site, plants could not be collected because their inhabiting plots were disturbed by construction activities when other plants were sampled. Four of the six collected species are annual or biennial plants (Table S3). They were in the reproductive stage at the time of collection.

### 2.2 Experimental Design of a Growth‐Chamber Study

To evaluate competitive ability of *L. serriola*, we chose three competing species commonly found in the invaded plots of field sites: *Chenopodium album*, *Erigeron canadensis*, and *Oenothera biennis*. All of these species are annual or biennial weeds. The origin of *C. album* is Eurasia, including South Korea, while the origin of *E. canadensis* and *O. biennis* is North or South America. *E. canadensis* and *O. biennis* were introduced to South Korea in the late 19th century and inhabit throughout the country now (Lee 2003). Seeds of those species were collected at the Seosan site. Due to insufficient seed numbers of *C. album*, seeds from the Yeongcheon site were also utilized. Similarly, we used *L. serriola* seeds from the Seosan and Gwangyang sites. Seeds from different sites were mixed together before sowing.

Seeds of each species were sown into plastic propagation trays filled with commercial soil medium (ShinSung Mineral Co., Kyeonggi‐do, Korea) and maintained for 2 months in a growth chamber at 22°C under a 16/8 h light/dark photoperiod with 200 μmol s^−1^ m^−2^ photosynthetically active radiation (PAR) intensity. Seedlings with three to five true leaves were transplanted into individual plastic pots (8 cm × 7.5 cm × 6 cm) containing vermiculite and sand in a volume ratio of 3:1.

One seedling of each testing species was randomly assigned to one of three competition treatments: control, interspecific, and intraspecific competition. In the control treatment, a single seedling was planted in a pot. In the intraspecific competition treatment, two seedlings of the same species were planted in a pot. Each pot in the interspecific competition treatment contained one seedling of testing species and one *L. serriola* seedling. 20 pots were prepared for the control, and 40 pots were prepared for each of the intraspecific and interspecific competition treatments for each species. Additionally, control and intraspecific competition pots were also prepared for *L. serriola*.

To assess the effects of soil nutrients on the competitive interactions, two nutrient treatments were implemented. For the low‐nutrient treatment, 20 mL of 0.1× Hoagland's solution was applied once a week for a month [1× Hoagland's solution, containing 1.25 mM KNO_3_, 1.5 mM Ca(NO_3_)_2_, 0.75 mM MgSO_4_, 0.5 mM KH_2_PO_4_, 0.05 mM H_3_BO_3_, 0.01 mM MnCl_2_, 0.002 mM ZnSO_4_, 0.0015 mM CuSO_4_, 0.075 μM NH_4_Mo_7_O_24_, and 0.074 mM Fe‐EDTA]. The high‐nutrient treatment received the same volume of 10× Hoagland's solution. The concentrations of available nitrogen and phosphorus in the high and low‐nutrient treatments fell within the range observed in the Seosan, Yeongcheon, and Gwangyang sites (S. Woo, unpublished data).

The pots were randomly positioned in a growth chamber set under the same conditions used for seed germination. They were watered with 20 mL of deionized water twice a week. Within 2 weeks of transplanting, one *O. biennis* plant (1 for control/low nutrient treatment) and 39 *E. canadensis* plants (3 for control/high nutrient, 7 for intraspecific/high nutrient, 7 for intraspecific/low nutrient, 11 for interspecific/high nutrient, and 10 for interspecific/low nutrient treatments) died, resulting in a total of 321 pots maintained until the end of the experiment. The number of replicates for each species and treatment is provided in Table S4.

1 month after transplanting, all plants were collected. A fully expanded leaf was collected from each individual plant separately to measure SLA. Leaves were photographed, and their surface areas were quantified using the ImageJ program (Schneider, Rasband, and Eliceiri 2012). Leaf dry weight was measured after drying the leaves at 65°C for 72 h (Garnier et al. 2001). SLA was calculated as the leaf area divided by the dry weight. Whole plant materials were washed with water and dried at 65°C for 72 h to measure aboveground and belowground dry weights. The RS ratio was calculated by dividing the dry weight of the roots by the dry weight of the shoots.

To evaluate the intensity of interspecific and intraspecific competition, the logarithmic response ratio (lnRR) was calculated using dry biomass (Goldberg et al. 1999; Tesfay, Blaschke, and Kreyling 2023). The formula used was:

$$\ln\mathrm{RR}=\left(B_{\text{cont}}/B_{\mathrm{mix}}\right)$$

where *B*_cont_ is the mean biomass of the target species grown alone, and *B*_mix_ represents the biomass of target species grown with a neighbor of the same species (intraspecific competition) or with a neighbor of different species (interspecific competition).

### 2.3 Statistical Analysis

All statistical analyses were conducted in R software 4.0.3. To compare species diversity indices, species richness, and soil characteristics between invaded and uninvaded plots, mixed model analyses of variance (ANOVA) were conducted using the *lme4* and *car* packages. The model included the invasion of *L. serriola* as a fixed factor and the site as a random factor. To examine plant biomass, the model included invasion of *L. serriola*, plant species, and their interaction as fixed factors, with the site as a random factor. *Post hoc* analyses were conducted using the Tukey method.

To assess the competitive ability of *L. serriola*, two separate analyses were conducted for the growth chamber experiment. First, considering the experimental design for the pairwise comparison, the entire dataset for the growth‐chamber study was divided into three: *L. serriola*—*C. album*, *L. serriola*—*O. biennis*, and *L. serriola*—*E. canadensis* datasets. For each dataset, three‐way ANOVA were conducted to examine plant traits among testing species, competition treatment (control, interspecific competition, intraspecific competition), nutrient treatment, and their interactions. Traits included total biomass, shoot and root biomass, RS ratio, and SLA. To interpret significant nutrient × species and nutrient × species × competition interactions (Table S6), the effect of competition and species were evaluated in each nutrient condition using *post hoc* Tukey method. Total biomass and shoot and root biomass were square root‐transformed, and SLA was log‐transformed to meet the normality assumption. The lnRRs between species and nutrient treatments were compared using two‐way ANOVA for each of three species‐pair datasets. The model included species and nutrient treatment as fixed factors. The lnRRs for intraspecific and interspecific competition were examined separately.

Second, the competitive responses of *L. serriola* to different competitor species under nutrient treatments were evaluated using a two‐way ANOVA. The dataset included pots for interspecific competition. The model included competing species, nutrient treatment, and their interactions. To interpret significant species × nutrient interactions (Table S8), the effect of competing species was evaluated in each nutrient condition using the *post hoc* Tukey method.

## 3 Results

### 3.1 Field Survey

**FIGURE 2:**
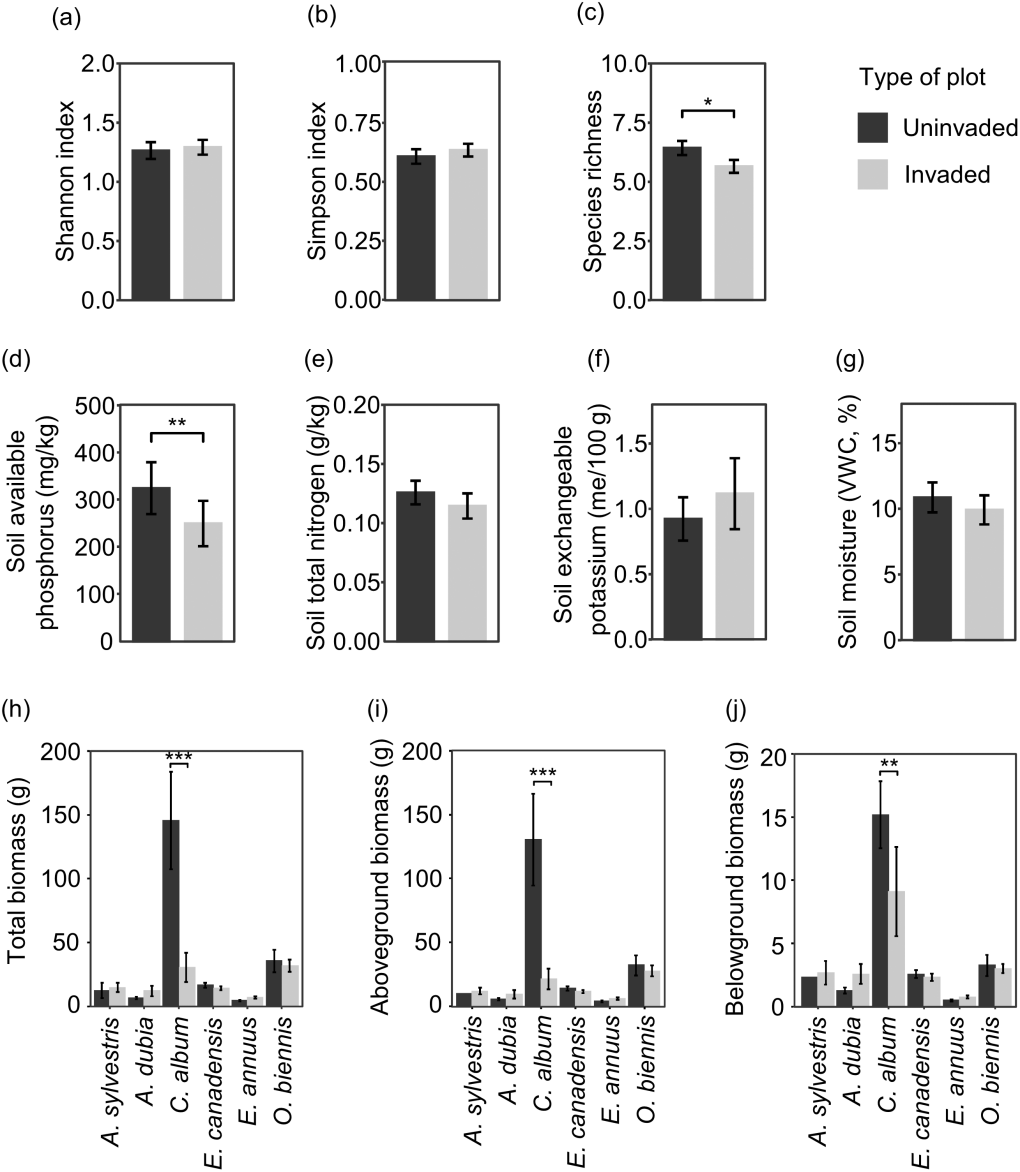
Species diversity indices, soil characteristics, and plant biomass in *Lactuca serriola* invaded and uninvaded plots. Averages and standard errors of the Shannon index (a), Simpson index (b), species richness (c), soil available phosphorus (d), soil total nitrogen (e), soil exchangeable potassium (f), soil moisture (g), total biomass (h), aboveground biomass (i), and belowground biomass (j) are provided. Asterisks in plant biomass indicate results of *post hoc* Tukey's multiple comparison tests. The full names of plant species are listed in Table S3, and the results of the analyses of variance for the biomass are presented in Table S5. *p < 0.05, **p < 0.01, ***p < 0.001.

The Shannon index and Simpson index of the plant communities did not differ between invaded and uninvaded plots (Shannon index, *F* = 0.156, *p* = 0.694; Simpson index, *F* = 0.843, *p* = 0.361) (Figure 2a,b), while the species richness in the invaded plots was slightly lower than that in the uninvaded plots (*F* = 4.921, *p* < 0.05) (Figure 1c). Soil of invaded plots exhibited a lower available phosphorus concentration than soil of uninvaded plots, although other soil characteristics measured were similar across invaded and uninvaded plots (Figure 2d–g). Overall, most plant species exhibited similar biomass in invaded and uninvaded plots (*Anthriscus sylvestris*, *t* = −1.599, *p* = 0.111; *Artemisia dubia*: *t* = −1.347, *p* = 0.179; *E. canadensis*: *t* = 0.523, *p* = 0.601; *Erigeron annuus*: *t* = −1.442, *p* = 0.150; *O. biennis*: *t* = −0.683, *p* = 0.495), while the biomass of *C. album* was lower in invaded plots compared to uninvaded plots (*t* = 3.450, *p* < 0.001) (Figure 2h–j, Table S5). Similar patterns were observed for aboveground and belowground biomass of plants.

### 3.2 Pair‐Wise Comparison of Competitive Ability

**FIGURE 3:**
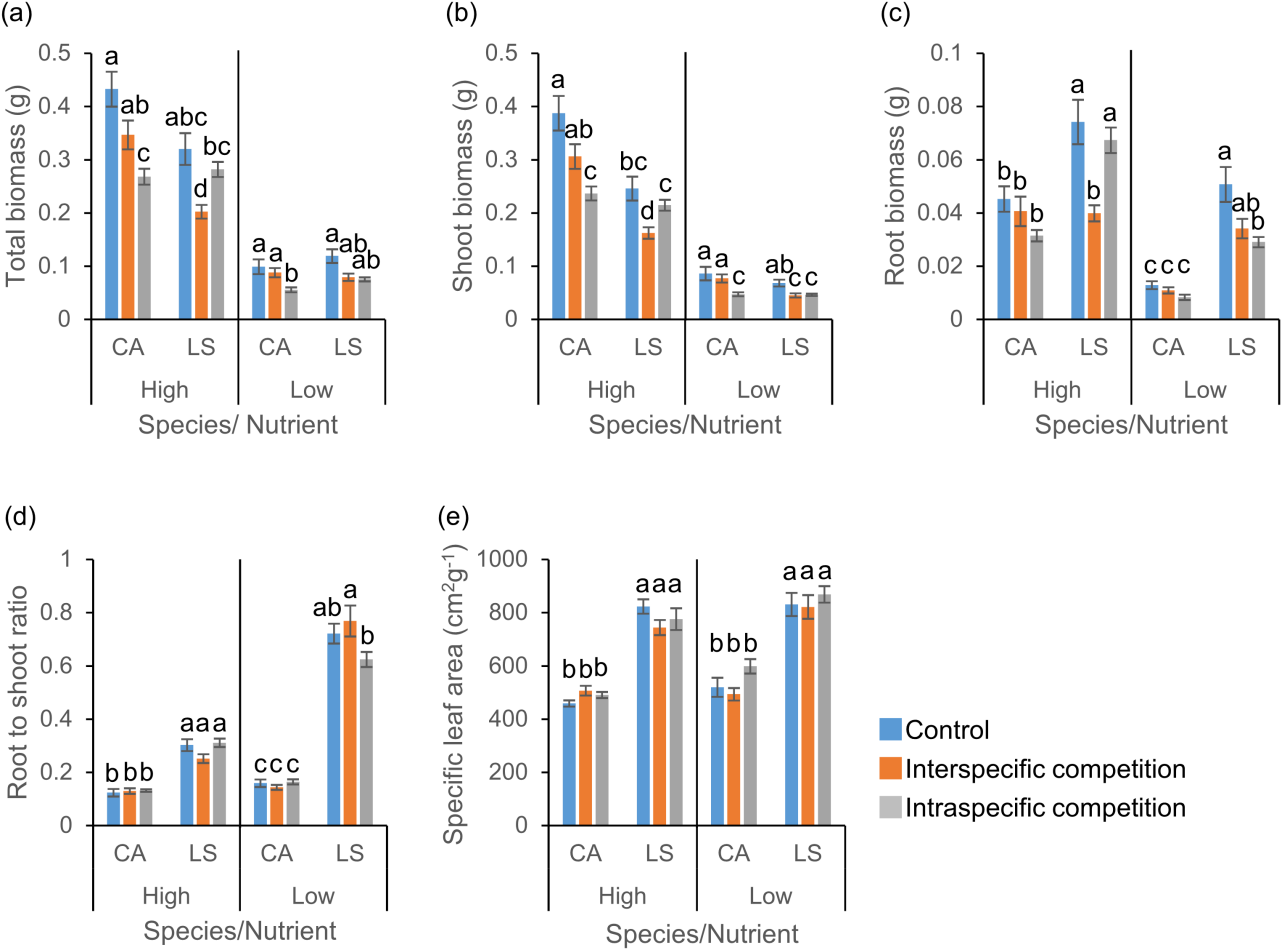
Effects of competition and nutrient treatments on traits of *Chenopodium album* and *Lactuca serriola*. Averages and standard errors for total biomass (a), shoot biomass (b), root biomass (c), root to shoot ratio (d), and specific leaf area (e) are provided. Letters indicate statistically significant differences at the 0.05 level based on Tukey's adjustment. See Table S6 for the results of analyses of variance. CA, *C. album*; LS, *L. serriola*.

When *C. album* and invasive *L. serriola* were examined, the competition treatment affected their total, aboveground, and belowground biomass (Table S6). Moreover, significant competition × species interactions and competition × species × nutrient treatment interactions were found (Table S6), indicating that competition effect on the biomasses differed between species and nutrient treatments. The total biomass decreased when *C. album* was grown with another plant in a pot compared to the biomass of plant without any neighboring plant (Figure 3a). Notably, *C. album* produced greater total biomass when grown with *L. serriola* rather than when grown with another *C. album* individual, regardless of nutrient conditions (Figure 3a). In contrast, under the high‐nutrient treatment, the total biomass of *L. serriola* in intraspecific competition was similar to the biomass in control and significantly higher than the biomass in interspecific competition (Figure 3a). Such patterns disappeared under the low‐nutrient treatment, with no significant differences in the total biomass of *L. serriola* between intra‐ and interspecific competition.

Aboveground and belowground biomass of *C. album* and *L. serriola* showed a similar trend as the total biomass, but statistical significances were slightly different (Figure 3b,c). The RS ratios (*F*_species_ = 497.26, *p* < 0.001) and SLA (*F*_species_ = 149.02, *p* < 0.001) of *L. serriola* were higher than those of *C. album* regardless of nutrient conditions (Figure 3d,e). The RS ratio of *L. serriola* slightly decreased in interspecific competition compared to that in intraspecific competition under low‐nutrient treatment (Figure 3d).

**FIGURE 4:**
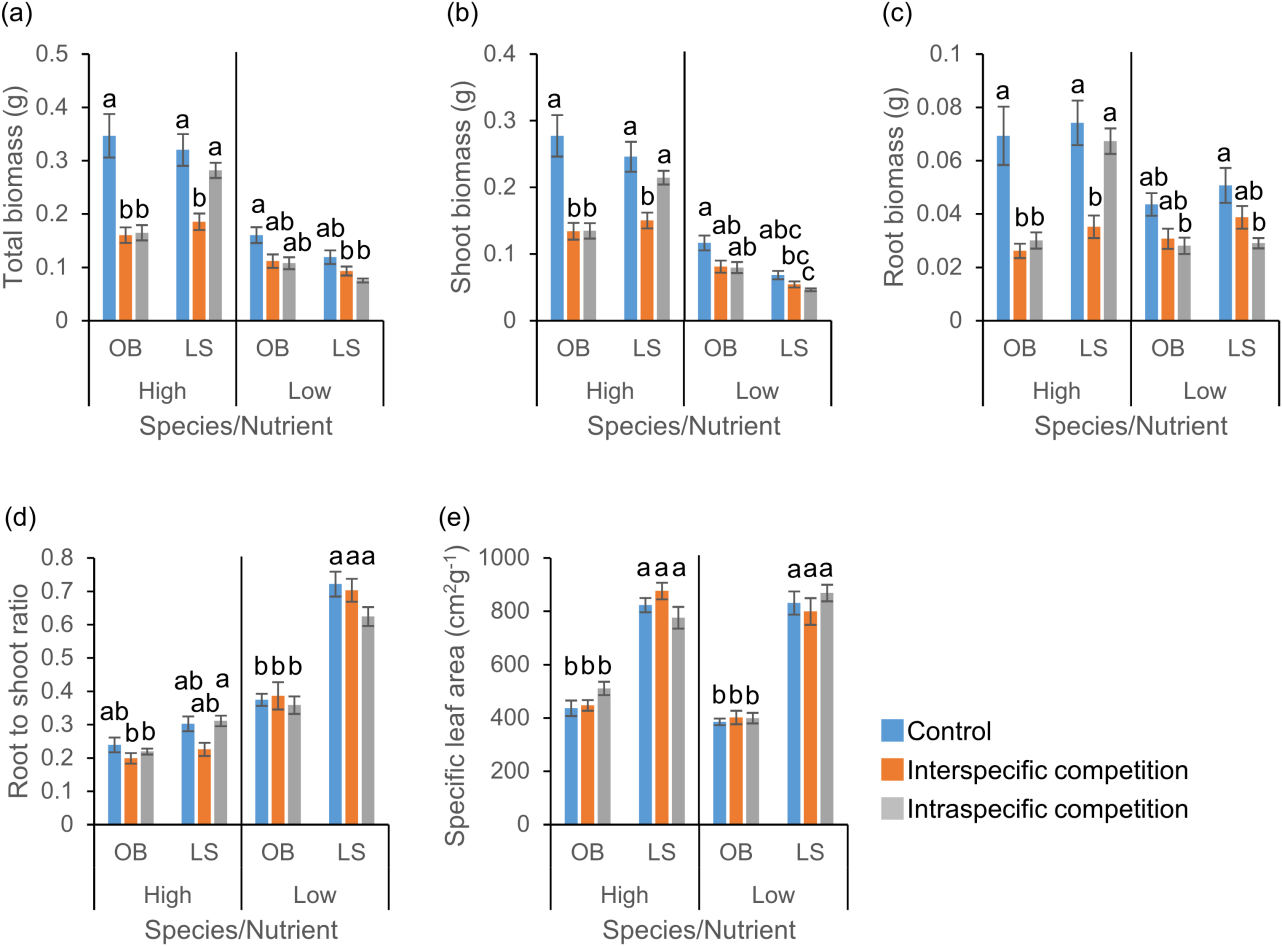
Effects of competition and nutrient treatments on traits of *Oenothera biennis* and *Lactuca serriola*. Averages and standard errors for total biomass (a), shoot biomass (b), root biomass (c), root to shoot ratio (d), and specific leaf area (e) are provided. Letters indicate statistically significant differences at the 0.05 level based on Tukey's adjustment. See Table S6 for the results of analyses of variance. OB, *O. biennis*; LS, *L. serriola*.

When grown with invasive *L. serriola*, *O. biennis* exhibited lower total, aboveground, and belowground biomass than those when it was grown alone (Figure 4a–c). Unlike *C. album*, the biomasses of *O. biennis* were similar in the interspecific and intraspecific competition under both high and low‐nutrient conditions. Similar to growth with *C. album*, *L. serriola* produced lower biomass when grown with *O. biennis* than when grown alone or with a conspecific individual under the high‐nutrient treatment (Figure 4a–c). No significant difference in biomass was detected between intraspecific and interspecific competition under the low‐nutrient treatment. *L. serriola* exhibited a higher RS ratio under the low‐nutrient treatment (Figure 4d) and a higher SLA in both nutrient conditions (Figure 4e) compared to *O. biennis*. Competition did not result in significant differences in the RS ratio and SLA.

**FIGURE 5:**
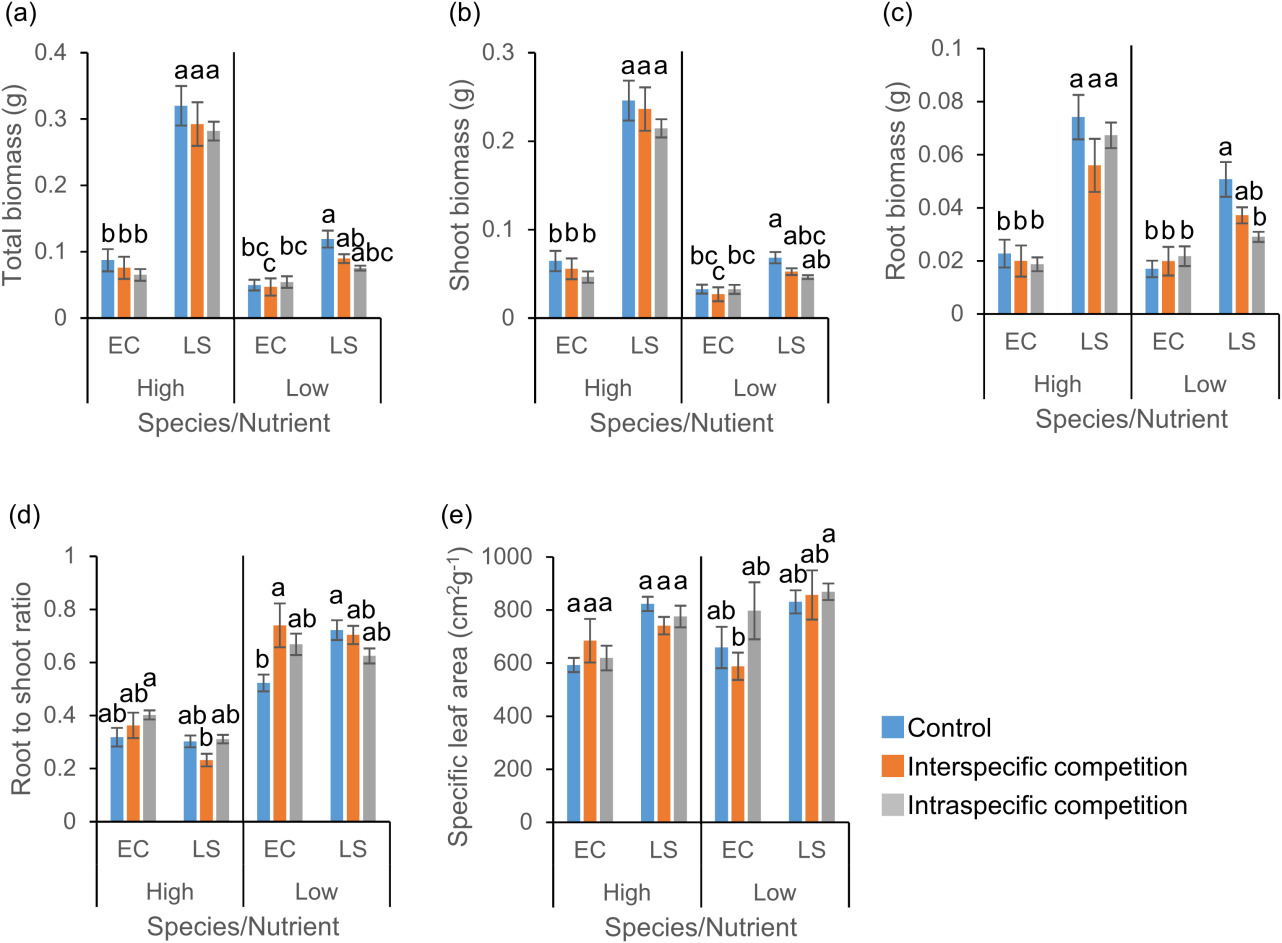
Effects of competition and nutrient treatments on traits of *Erigeron canadensis* and *Lactuca serriola*. Averages and standard errors for total biomass (a), shoot biomass (b), root biomass (c), root to shoot ratio (d), and specific leaf area (e) are provided. Letters indicate statistically significant differences at the 0.05 level based on Tukey's adjustment. See Table S6 for the results of analyses of variance. EC, *E. canadensis*; LS, *L. serriola*.

*E. canadensis* produced similar total, aboveground, and belowground biomass across competition treatments (Figure 5a–c). When grown with *E. canadensis*, *L. serriola* tended to produce slightly lower biomasses than those grown alone, but the differences were not statistically significant (Figure 5a–c). *L. serriola* exhibited a similar RS ratio and SLA compared to *E. canadensis* (Figure 5d,e).

### 3.3 Intensity of Interspecific and Intraspecific Competition

**FIGURE 6:**
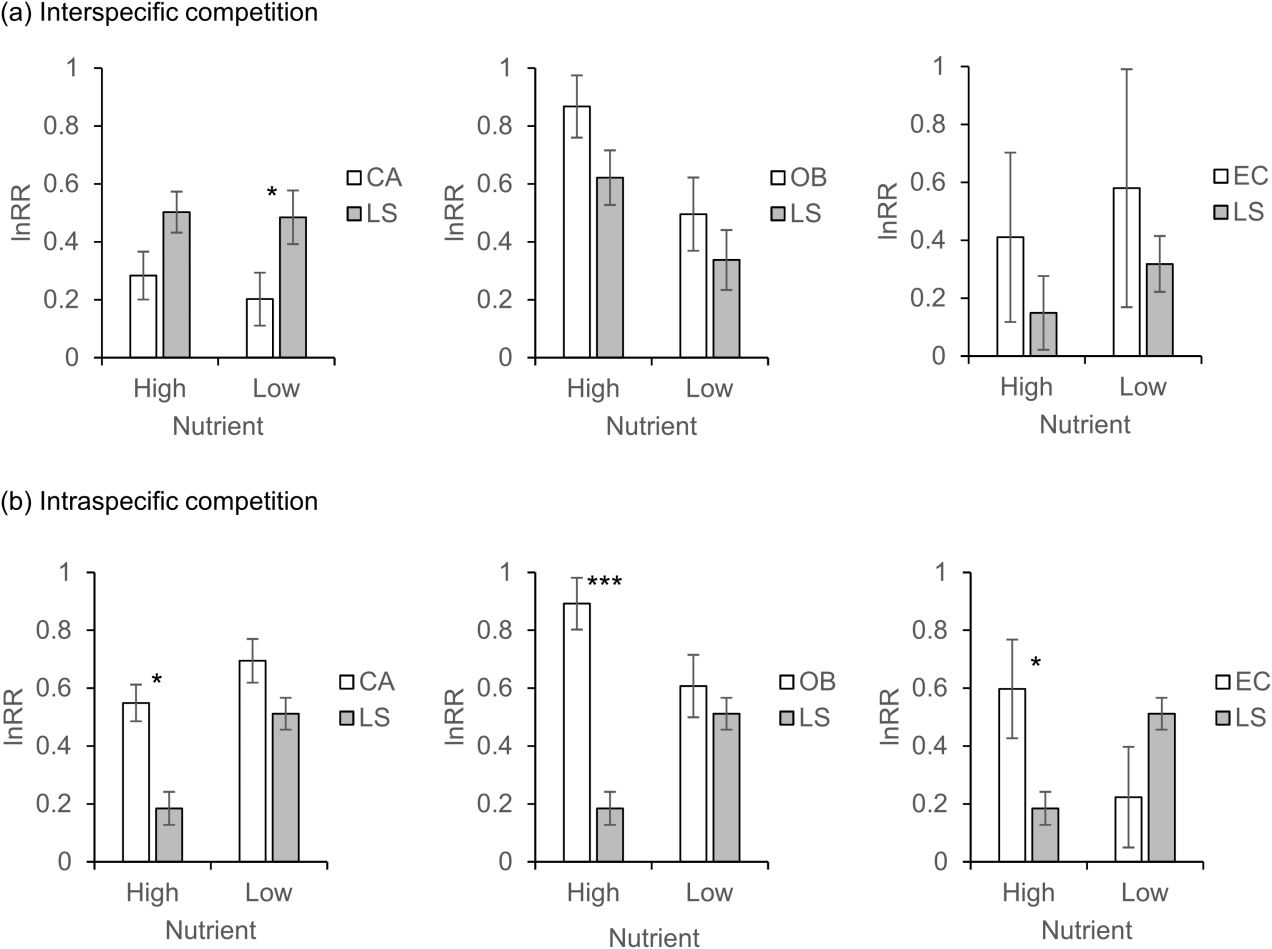
The logarithmic response ratios (lnRR) for the interspecific (a) and intraspecific competition (b) traspecific competition in the high and low nutrient treatments. Averages and standard errors are given. The entire dataset was divided into three datasets (*Lactuca serriola*—*Chenopodium album*, *L. serriola*—*Oenothera biennis*, and *L. serriola*—*Erigeron canadensis* datasets) and analyzed separately. Results of analyses of variance are presented in Table S7. CA, *C. album*; EC, *E. canadensis*; LS, *L. serriola*; OB, *O. biennis*. *p < 0.05, ***p < 0.001.

The intensity of interspecific competition between invasive *L. serriola* and co‐occurring plant species varied among species pairs (Table S7). The competitive effect of *L. serriola* on *C. album* was weaker than the effect of *C. album* on *L. serriola* (*F* = 8.77, *p* < 0.01), which was manifest in the low‐nutrient treatment (Figure 6a). In contrast, no significant difference in interspecific lnRR was detected in the *O. biennis*—*L. serriola* and *E. canadensis*—*L. serriola* pairs (Figure 6a). In the *O. biennis*—*L. serriola* pair, low‐nutrient treatment reduced the intensity of interspecific competition (*F* = 9.12, *p* < 0.01) (Figure 6a).

The intensity of intraspecific competition showed significant species by nutrient interactions, indicating that the difference between invasive and co‐occurring species depended on nutrient conditions (Table S7). Invasive *L. serriola* exhibited weaker intraspecific lnRR than other species in the high‐nutrient treatment (Figure 6b). However, such a difference was not found in the low‐nutrient treatment (Figure 6b).

### 3.4 Competitive Response of *L. serriola* to Different Competitor Species

**FIGURE 7:**
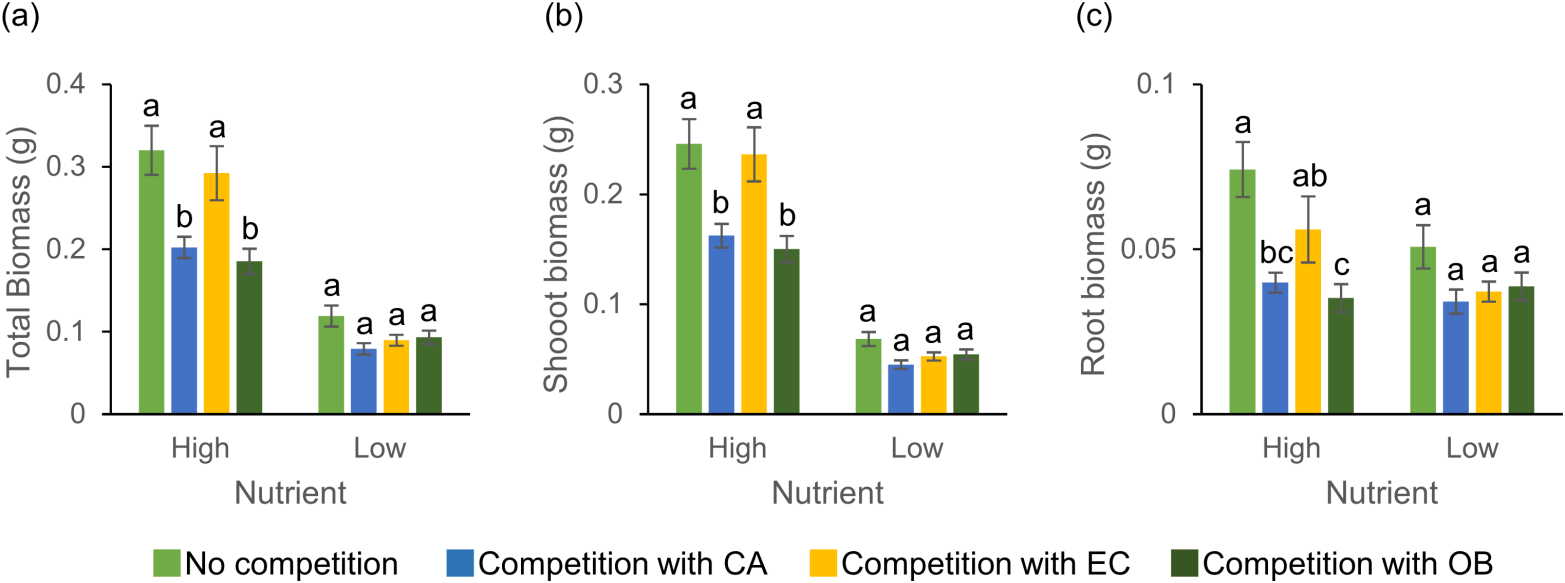
Performance of *Lactuca serriola* in response to competition and nutrient treatments. Averages and standard errors of *L. serriola* traits are provided. Letters indicate statistically significant differences at the 0.05 level based on Tukey's adjustment. See Table S8 for the results of the analyses of variance. CA, *Chenopodium album*; EC, *Erigeron canadensis*; OB, *Oenothera biennis*.

Both competitor identity and nutrient treatments affected the growth of invasive *L. serriola* (Table S8). The total biomass of *L. serriola* grown with *E. canadensis* was similar to that of control plants without a competitor (Figure 7a). In contrast, when *L. serriola* was grown with *C. album* or *O. biennis*, its total biomass decreased compared to the control under high‐nutrient treatment (Figure 7a). Such differences were not detected under low nutrient treatment. Similar patterns were observed for the shoot and root biomass of *L. serriola* (Figure 7b,c; Table S8). Competitor identity did not influence the RS ratio (*F*_species_ = 0.99, *p* = 0.40) or SLA (*F*_species_ = 0.87, *p* = 0.45) of *L. serriola* (Table S8).

## 4 Discussion

### 4.1 Interspecific Competition in *L. Serriola* and Co‐Occurring Plant Species

While *L. serriola* has rapidly spread throughout South Korea, we found no evidence indicating its competitive superiority over co‐occurring weed species. In the field, diversity indices and the biomass of co‐occurring plants were similar across plots with and without *L. serriola* (Figure 2). In a controlled environment, when three weed species (*C. album*, *O. biennis*, and *E. canadensis*) were grown with *L. serriola*, they produced similar or greater biomass compared to those with a conspecific individual, regardless of nutrient conditions (Figures 3, 4, 5). Moreover, lnRR indices of interspecific competition for testing weed species were lower or comparable to those for *L. serriola* (Figure 6). These observations diverge from the general expectation that invasive species negatively impact recipient communities due to their superior competitive abilities (Gaertner et al. 2009; Jauni, Gripenberg, and Ramula 2015; Sakai et al. 2001; Vilà and Weiner 2004). Instead, our results support the hypothesis that invasive plants possess similar competitive abilities as common plants in the introduced area (Dawson, Fischer, and van Kleunen 2012; Zhang and van Kleunen 2019).

Nutrient conditions are suggested to influence competitive ability (Gioria and Osborne 2014). Invasive plants typically demonstrate competitive advantages in high‐nutrient environments, but native plants often exhibit higher competitive ability than alien plants in low‐nutrient environments (Daehler 2003). In contrast, several case studies report that some invasive plants adopt the high root allocation strategies in low‐nutrient environments, potentially mitigating the impact of nutrient shortage and sustaining their competitiveness (Funk 2008; Grotkopp and Rejmánek 2007). Our field survey revealed that the available phosphorous in soil is lower in *L. serriola*‐invaded plots than in uninvaded plots (Figure 2). In addition, *L. serriola* exhibited a higher RS ratio compared to *C. album* and *O. biennis*, with this pattern being more pronounced in the low‐nutrient treatment (Figures 3, 4). Considering these results, we anticipated that the competitive ability of *L. serriola* might depend on soil nutrient conditions.

However, when interspecific lnRRs were examined, no significant nutrients by species interactions were found in all species pairs (Table S7), indicating that the hierarchy of the interspecific competition effect was maintained across the tested nutrient conditions (Figure 6). Despite this result, it should be noted that we only examined the competitive intensity of plants at the early developmental stage. The competitive advantage of invasive plants can become more apparent at a later developmental stage because the rapid growth rate of invasive plants could result in a large size with a higher resource uptake capacity (Mangla et al. 2011). Considering a larger SLA and RS ratio observed in *L. serriola* (Figures 3, 4) compared to co‐occurring plant species and their potential contribution to the plant growth rate (Grotkopp and Rejmánek 2007; van Kleunen, Bossdorf, and Dawson 2018), conducting a long‐term experiment is required for drawing a more comprehensive conclusion regarding the interspecific competition abilities.

Some alien plants with low competitive ability are known to successfully invade the introduced areas. For instance, disturbance or fluctuating environmental conditions can mitigate competitive intensity between invaders and natives (Davis, Grime, and Thompson 2000; Lembrechts et al. 2016; Liu, Yang, and Zhu 2018; Seabloom et al. 2003), thereby facilitating invasive success. Invasive species with a low competitive effect may exhibit higher tolerance to environmental stresses (Tesfay, Blaschke, and Kreyling 2023) or greater plasticity in functional traits, contributing to their establishment in the introduced area (Fagúndez and Lema 2019). Given the higher RS ratio of *L. serriola* compared to testing weed plant species (Figures 3, 4) and its well‐known drought resistance (Chadha and Florentine 2021), it is plausible that high tolerance to drought stress contributes to the invasive success of *L. serriola*.

### 4.2 Intraspecific Competition in *L. Serriola* and Co‐Occurring Plant Species

The reduction in biomass of *L. serriola* due to intraspecific competition was significantly smaller than that resulting from interspecific competition (Figures 3, 4), and the intensity of intraspecific competition for *L. serriola* was lower than that observed in testing weed plant species (Figure 6). This is in contrast to the longstanding assumption that intraspecific competition between invasive individuals would be predominant compared to interspecific competition with neighboring plants, likely due to superior competitive ability of invasive plants to plants in the introduced area (Gioria and Osborne 2014, and references therein). While this phenomenon has not been widely acknowledged in invasive biology, plant species with weak interspecific competitive effects can coexist with others, exhibiting stronger interspecific competitive effects if they have low intraspecific competition intensity, enabling the establishment of high conspecific density (Barabás, Michalska‐Smith, and Allesina 2016; Wassmuth et al. 2009). Given the significant contribution of intraspecific competition to the outcome of competitive interactions (Hart, Freckleton, and Levine 2018), the low intraspecific competition would provide an additional advantage for invasion of *L. serriola*, contributing to its successful establishment in the introduced area (Bossdorf et al. 2004; Holway, Suarez, and Case 1998).

The extent to which low intraspecific competition is a common characteristic among successful invasive species remains uncertain. One study showed that genotypes of invasive species from native areas tend to outcompete those from the introduced areas (Bossdorf et al. 2004). Additionally, intraspecific competition between genotypes from introduced areas has a lesser impact on biomass than that observed among genotypes from native areas (Zhang et al. 2019). Zhang et al. (2019) suggested that the genetic diversity in the introduced area is typically smaller than that of native area during the early stages of invasion, potentially leading to kin selection favoring a reduced competitive effect among conspecific individuals. Since we lack genotype information for our test plants, it remains uncertain whether kin selection operates in *L. serriola*.

Lower lnRR values of invasive *L. serriola* were evident only in the high‐nutrient treatment, with no statistically significant difference detected in the low‐nutrient treatment. Similar to interspecific competition, intraspecific competition would also depend on nutrient conditions. This implies that ecological advantages of low intraspecific competition would be manifest in fertile environments. In disturbed areas with high nutrient, like fallow farmland, *L. serriola* seedlings could quickly establish dense populations, potentially serving as a source population dispersing to other areas through highly dispersible seeds (achenes with pappus).

## 5 Conclusions

In this study, consistent results from field surveys and a growth‐chamber experiment revealed that invasive *L. serriola* is not a superior competitor suppressing co‐occurring plant species in invaded communities. The diversity indices of plant communities in the invaded plots were similar to those in the uninvaded plots, though species richness was slightly lower at nine study sites. The interspecific competitive effect of *L. serriola* was smaller than or similar to those of co‐occurring weedy plants. Notably, *L. serriola* exhibited weak intraspecific competitive intensity compared to tested weedy plant species at least in the early developmental stage, which could potentially contribute to its establishment in the introduced area. While intraspecific competition has been largely ignored in invasive biology, more studies are required to evaluate its role in invasive success, as intraspecific competitive intensity could influence invasive dynamics (Bossdorf et al. 2004; Gioria and Osborne 2014).

## Author Contributions

**Sohyun Woo:** conceptualization (lead), data curation (lead), formal analysis (lead), investigation (lead), methodology (lead), visualization (lead), writing – original draft (lead). **Tae‐Min Kim:** data curation (equal), investigation (equal). **Yousuk Kim:** data curation (equal), investigation (equal). **Seorin Jeong:** data curation (equal), investigation (equal). **Eunsuk Kim:** conceptualization (lead), data curation (equal), formal analysis (equal), funding acquisition (lead), investigation (lead), methodology (lead), supervision (lead), validation (lead), writing – original draft (equal), writing – review and editing (lead).

## Conflicts of Interest

The authors declare no conflicts of interest.

## Supporting information


**Table S1.** Locations of study sites.
**Table S2.** Plant species found in both *Lactuca serriola* invaded and uninvaded plots in nine study sites (Figure 1). Origin and life history characteristics of species are given based on the National Species Information System (http://www.nature.go.kr/main/Main.do) and Information of Korean Alien Species (https://kias.nie.re.kr/home/main/main.do).
**Table S3.** Dominant weedy plant species in testing sites. Numbers in parentheses indicate the number of plant samples collected to measure biomass from uninvaded plots and the number of plant samples collected from invaded plots. The names of sites are given in Figure 1.
**Table S4.** Number of pots used in the growth‐chamber study. CA, *Chenopodium album*; OB, *Oenothera biennis*; EC, *Erigeron canadensis*; LS, *Lactuca serriola*.
**Table S5.** Results of the analysis of variance (ANOVA) comparing the dry biomass of co‐occurring plants between *Lacuca serriola* invaded and uninvaded plots across study sites. The ANOVA model included plots, test species, and their interaction as fixed factors, with a study site as a random factor. F‐ratios are given. *p < 0.05, **p < 0.01, ***p < 0.001.
**Table S6.** Results of analyses of variance comparing plant traits among plant species, competition, and nutrient treatments. Considering the experimental design for the pairwise comparison, the entire dataset for the growth‐chamber study was divided into three: *Lactuca serriola*—*Chenopodium album* (CA‐LS), *L. serriola*—*Oenothera biennis* (OB‐LS), and *L. serriola*—*Erigeron canadensis* (EC‐LS) datasets. F‐ratios are given. *p < 0.05, **p < 0.01, ***p < 0.001.
**Table S7.** Results of analyses of variance comparing the logarithmic response ratio (lnRR) for interspecific and intraspecific competition among plant species and nutrient treatments. Considering the experimental design for the pairwise comparison, the entire dataset for the growth‐chamber study was divided into three: *Lactuca serriola*—*Chenopodium album* (CA‐LS), *L. serriola*—*Oenothera biennis* (OB‐LS), and *L. serriola*—*Erigeron canadensis* (EC‐LS) datasets.F‐ratios are given. **p < 0.01, ***p < 0.001.
**Table S8.** Results of analysis of variance comparing competitive responses to different competitor species under nutrient treatments. The dataset included plots for interspecific competition in the growth‐chamber study. F‐ratios are given. *p < 0.05, **p < 0.01, ***p < 0.001.

## Data Availability

The dataset and R code for statistical analysis is available in Figshare: https://doi.org/10.6084/m9.figshare.25856860.
