# Supplementary material for: Inter‐ and Intraspecific Competition in Invasive Lactuca serriola and Co‐Occurring Weedy Plant Species
Source: Ecol Evol. 2024 Oct 29;14(10):e70496. doi: 10.1002/ece3.70496 (PMC11522139; doi:10.1002/ece3.70496)
Supplement: Supplementary file 1 — Table S1. Locations of study sites. Table S2. Plant species found in both Lactuca serriola invaded and uninvaded plots in nine study sites (Figure 1). Origin and life history characteristics of species are given based on the National Species Information System (http://www.nature.go.kr/main/Main.do) and Information of Korean Alien Species (https://kias.nie.re.kr/home/main/main.do). Table S3. Dominant weedy plant species in testing sites. Numbers in parentheses indicate the number of plant samples collected to measure biomass from uninvaded plots and the number of plant samples collected from invaded plots. The names of sites are given in Figure 1. Table S4. Number of pots used in the growth‐chamber study. CA, Chenopodium album; OB, Oenothera biennis; EC, Erigeron canadensis; LS, Lactuca serriola. Table S5. Results of the analysis of variance (ANOVA) comparing the dry biomass of co‐occurring plants between Lacuca serriola invaded and uninvaded plots across study sites. The ANOVA model included plots, test species, and their interaction as fixed factors, with a study site as a random factor. F‐ratios are given. *p < 0.05, **p < 0.01, ***p < 0.001. Table S6. Results of analyses of variance comparing plant traits among plant species, competition, and nutrient treatments. Considering the experimental design for the pairwise comparison, the entire dataset for the growth‐chamber study was divided into three: Lactuca serriola—Chenopodium album (CA‐LS), L. serriola—Oenothera biennis (OB‐LS), and L. serriola—Erigeron canadensis (EC‐LS) datasets. F‐ratios are given. *p < 0.05, **p < 0.01, ***p < 0.001. Table S7. Results of analyses of variance comparing the logarithmic response ratio (lnRR) for interspecific and intraspecific competition among plant species and nutrient treatments. Considering the experimental design for the pairwise comparison, the entire dataset for the growth‐chamber study was divided into three: Lactuca serriola—Chenopodium album (CA‐LS), L. serriola—Oenoth [file ECE3-14-e70496-s001.docx]

Table S1. Locations of study sites.

| Site | Location | latitude | longitude |
| --- | --- | --- | --- |
| AD | Andong, Gyeongsangbuk-do | 36.55045 | 128.7842 |
| CHA | Cheonan, Chungcheongnam-do | 36.70856 | 127.1183 |
| GCH | Gimcheon, Gyeongsangbuk-do | 36.13695 | 128.1487 |
| GJ | Gwangju | 35.24265 | 126.8731 |
| GY | Gwangyang, Jeollanam-do | 34.9414 | 127.7112 |
| JS | Jeongseon, Gangwon-do | 37.21366 | 128.6452 |
| SS | Seosan, Chungcheongnam-do | 36.80966 | 126.5793 |
| TB | Taebaek, Gangwon-do | 37.09552 | 129.0281 |
| YCH | Yeongcheon, Gyeongsangbuk-do | 35.9082 | 129.0127 |

Table S2. Plant species found in both *L. serriola* invaded and uninvaded plots in nine study sites (Fig. 1). Origin and life history characteristics of species are given based on the National Species Information System (http://www.nature.go.kr/main/Main.do) and Information of Korean Alien Species (https://kias.nie.re.kr/home/main/main.do).

| Species | Origin | Life history characteristics |
| --- | --- | --- |
| *Artemisia dubia* | East Asia | Perennial, flowering from August to September |
| *Chenopodium album* | Eurasia | Annual, flowering from June to July |
| *Elymus tsukushiensis* | East Asia | Perennial, flowering in July |
| *Oenothera biennis* | North America | Biennial, flowering from June to September |
| *Anthriscus sylvestris* | Eurasia and African Mountains | Perennial, flowering from May to June |
| *Bromus japonicus* | Eurasia | Annual, flowering in May |
| *Erigeron canadensis* | South and North America | Annual/Biennial, flowering from July to September |
| *Erigeron annuus* | North America | Annual/Biennial, flowering from June to July |
| *Vicia villosa* | North Africa, East Central and South Europe, and Central Asia | Annual/Biennial, flowering from May to June |

Table S3. Dominant weedy plant species in testing sites. Numbers in parentheses indicate the number of plant samples collected to measure biomass from uninvaded plots and the number of plant samples collected from invaded plots. The names of sites are given in Figure 1 and Table S1.

| Site | Dominant plant species |
| --- | --- |
| AD | *Erigeron canadensis* (9, 9) *Erigeron annuus* (9,9), *Oenothera biennis* (9,6) |
| CHA | *Artemisia dubia* (15,6), *E. annuus* (3,12), *O. biennis* (5,12) |
| GCH | *E. canadensis* (15, 15), *E. annuus* (15, 3), *O. biennis* (15, 10) |
| GJ | *A. dubia* (12, 3), *E. canadensis* (6, 9), *E. annuus* (15, 14) |
| GY | *E. canadensis* (9, 15), *E. annuus* (13, 10), *O. biennis* (12, 10) |
| JS | *Bromus japonicas* (0, 0), *E. canadensis* (15, 15), *Vicia villosa* (0, 0) |
| SS | *E. canadensis* (15, 15), *E. annuus* (6, 11), *O. biennis* (8, 15) |
| TB | *Anthriscus sylvestris* (9, 9), *Chenopodium album* (9, 5), *E. canadensis* (12, 11) |
| YCH | *C. album* (14, 3), *E. canadensis* (15, 15), *E. annuus* (9, 13) |

Table S4. Number of pots used in the growth-chamber study. CA, *Chenopodium album*; OB, *Oenothera biennis*; EC, *Erigeron canadensis*; LS, *Lactuca serriola*.

|  |  | High nutrient | Low nutrient |
| --- | --- | --- | --- |
| *Chenopodium album* | Control | 10 | 10 |
|  | Monoculture | 20 | 20 |
|  | CA-LS mixed culture | 20 | 20 |
| *Oenothera biennis* | Control | 10 | 9 |
|  | Monoculture | 20 | 20 |
|  | OB-LS mixed culture | 20 | 20 |
| *Erigeron canadensis* | Control | 7 | 10 |
|  | Monoculture | 13 | 13 |
|  | EC-LS mixed culture | 9 | 10 |
| *Lactuca serriola* | Control | 10 | 10 |
|  | Monoculture | 20 | 20 |

Table S5. Results of the analysis of variance (ANOVA) comparing the dry biomass of co-occurring plants between *Lacuca serriola* invaded and uninvaded plots across study sites. The ANOVA model included plots, test species, and their interaction as fixed factors, with study site as a random factor. F-ratios are given. * *P* < 0.05, ** *P* < 0.01, *** *P* < 0.001.

|  | Plot | Species | Plot × species |
| --- | --- | --- | --- |
| Total biomass | 0.060 | 51.37*** | 3.75** |
| Shoot biomass | 0.000 | 51.18*** | 3.97** |
| Root biomass | 0.000 | 41.84*** | 2.85* |

Table S6. Results of analysis of variance comparing plant traits among plant species, competition, and nutrient treatments. Considering the experimental design for the pairwise comparison, the entire dataset for the growth-chamber study was divided into three: *Lactuca serriola – Chenopodium album (CA-LS), L. serriola – Oenothera biennis (OB-LS), and L. serriola – Erigeron canadensis (EC-LS)* datasets*.*  F-ratios are given. * *P* < 0.05, ** *P* < 0.01, *** *P* < 0.001.

|  | Species | Compe- tition | Nutrient | Spec × Comp | Spec ×  Nut | Comp × Nut | Spec × Comp × Nut |
| --- | --- | --- | --- | --- | --- | --- | --- |
| CA-LS |  |  |  |  |  |  |  |
| Total biomass | 6.25* | 20.07*** | 636.27*** | 13.44*** | 19.62*** | 2.36 | 3.52* |
| Shoot biomass | 49.53*** | 20.29*** | 789.44*** | 15.35*** | 13.87*** | 2.04 | 2.24 |
| Root biomass | 135.87*** | 10.88*** | 149.21*** | 6.84** | 12.96*** | 3.94* | 5.71** |
| Root to shoot ratio | 497.26*** | 0.74 | 194.65*** | 1.86 | 149.83*** | 4.59* | 6.42** |
| Specific leaf area | 149.02*** | 0.78 | 4.82* | 1.23 | 0.01 | 2.1 | 0.7 |
| OB-LS |  |  |  |  |  |  |  |
| Total biomass | 0.11 | 23.18*** | 163.53*** | 4.25* | 13.72*** | 6.37** | 4.37* |
| Shoot biomass | 1.76 | 21.81*** | 249.97*** | 4.83** | 20.74*** | 5.36** | 3.53* |
| Root biomass | 15.75*** | 21.37*** | 15.32*** | 2.66 | 3.36 | 9.33*** | 5.16** |
| Root to shoot ratio | 105.15*** | 0.97 | 237.83*** | 0.23 | 47.51*** | 4.61* | 1.68 |
| Specific leaf area | 251.62*** | 0.01 | 3.4 | 0.94 | 4.94* | 0.36 | 3.95* |
| EC-LS |  |  |  |  |  |  |  |
| Total biomass | 203.77*** | 3.25* | 122.09*** | 0.61 | 50.39*** | 0.12 | 1.17 |
| Shoot biomass | 233.97*** | 3.76* | 206.86*** | 0.4 | 76.00*** | 0.64 | 0.67 |
| Root biomass | 111.20*** | 2.03 | 12.81*** | 1.45 | 10.06** | 0.46 | 2.11 |
| Root to shoot ratio | 0.67 | 1.01 | 200.56*** | 4.67* | 6.06* | 3.14* | 1.17 |
| Specific leaf area | 19.44*** | 0.11 | 1.44 | 0.45 | 0.23 | 1.66 | 0.73 |

Table S7. Results of analysis of variance comparing logarithmic response ratio (lnRR) for interspecific and intraspecific competition among plant species and nutrient treatments. Considering the experimental design for the pairwise comparison, the entire dataset for the growth-chamber study was divided into three: *Lactuca serriola – Chenopodium album (CA-LS), L. serriola – Oenothera biennis (OB-LS), and L. serriola – Erigeron canadensis (EC-LS)* datasets*.* F-ratios are given. ** *P* < 0.01, *** *P* < 0.001.

|  | Species | Nutrient | Species × nutrient |
| --- | --- | --- | --- |
| Interspecific competition |  |  |  |
| CA-LS | 8.77** | 0.34 | 0.14 |
| EC-LS | 0.94 | 0.4 | 0.00 |
| OB-LS | 3.46 | 9.12** | 0.16 |
| Intraspecific competition |  |  |  |
| CA-LS | 18.76*** | 14.00*** | 2.06 |
| EC-LS | 0.32 | 0.05 | 10.26** |
| OB-LS | 24.87*** | 0.07 | 14.43*** |

Table S8. Results of analysis of variance comparing competitive responses to different competitor species under nutrient treatments. The dataset included pots for interspecific competition in the growth-chamber study. F-ratios are given. * *P* < 0.05, ** *P* < 0.01, *** *P* < 0.001.

|  | Competing species | Nutrient | Competing species × nutrient |
| --- | --- | --- | --- |
| Total biomass | 10.50*** | 193.44*** | 3.78* |
| Shoot biomass | 10.36*** | 348.92*** | 4.24** |
| Root biomass | 8.74*** | 6.59* | 2.34 |
| Root to shoot ratio | 0.99 | 279.99*** | 0.52 |
| Specific leaf area | 0.87 | 0.26 | 2.09 |
